# Thyroid Hormone Signaling in Retinal Development and Function: Implications for Diabetic Retinopathy and Age-Related Macular Degeneration

**DOI:** 10.3390/ijms25137364

**Published:** 2024-07-04

**Authors:** Giuseppina Nicolini, Giovanni Casini, Chiara Posarelli, Rosario Amato, Matteo Lulli, Silvana Balzan, Francesca Forini

**Affiliations:** 1 CNR Institute of Clinical Physiology, 56124 Pisa, Italy; giuseppina.nicolini@cnr.it (G.N.); francesca.forini@cnr.it (F.F.); 2 Department of Biology, University of Pisa, 56127 Pisa, Italy; rosario.amato@unipi.it; 3 Ophthalmology, Department of Surgical, Medical and Molecular Pathology and Critical Care Medicine, University of Pisa, 56126 Pisa, Italy; chiara.posarelli@unipi.it; 4 Department of Experimental and Clinical Biomedical Sciences “Mario Serio”, University of Florence, 50134 Florence, Italy; matteo.lulli@unifi.it

**Keywords:** thyroid hormone signaling, diabetic retinopathy, age-related macular degeneration

## Abstract

Thyroid Hormones (THs) play a central role in the development, cell growth, differentiation, and metabolic homeostasis of neurosensory systems, including the retina. The coordinated activity of various components of TH signaling, such as TH receptors (THRs) and the TH processing enzymes deiodinases 2 and 3 (DIO2, DIO3), is required for proper retinal maturation and function of the adult photoreceptors, Müller glial cells, and pigmented epithelial cells. Alterations of TH homeostasis, as observed both in frank or subclinical thyroid disorders, have been associated with sight-threatening diseases leading to irreversible vision loss i.e., diabetic retinopathy (DR), and age-related macular degeneration (AMD). Although observational studies do not allow causal inference, emerging data from preclinical models suggest a possible correlation between TH signaling imbalance and the development of retina disease. In this review, we analyze the most important features of TH signaling relevant to retinal development and function and its possible implication in DR and AMD etiology. A better understanding of TH pathways in these pathological settings might help identify novel targets and therapeutic strategies for the prevention and management of retinal disease.

## 1. Introduction

Several lines of evidence point to a pivotal role of thyroid hormone (TH) signaling in controlling the development and function of neuro-sensorial organs, including the retina [1,2,3]. The main actions of TH signaling in retinal physiology deal with orchestration of post-natal differentiation and with the maintenance of mature retina tasks through the regulation of photoreceptor, Müller glial cell, and pigmented epithelial cell function [4,5,6,7].

It is worth noting that THs influence retinal maturation in a spatio-temporal dynamic during limited developmental time windows. For instance, TH deficiency during fetal and neonatal periods determines changes in neuronal migration and differentiation as well as a noticeable delay in photoreceptor development, impairments of eye growth, and alteration of visual system function [6,8]. In addition, a postnatal surge in the biologically active TH, 3,5′,3 triiodothyronine (T3), and upregulation of the T3-dependent expression program is required for the maturation and function of cone photoreceptors and cells of the retinal pigment epithelium. Previous data suggest that TH signaling may act early in development by stimulating specific cell populations and favoring cell–cell interactions in order to prompt normal growth of the target ocular tissues [6,7,9]. Overall, the available findings suggest that the TH axis could favor proper gene expression by retinal neuroblastic cell mass in order to acquire the definitive cell phenotypes and/or to make the correct connections through the retinal layering and the optic pathway; also, TH signaling could modulate neuron fate decisions, influencing retinal progenitor cells to make the adequate numbers and types of ganglion cells, photoreceptor cells, and other retinal cell types [6].

TH signaling is also critically involved in the maintenance of mature retina physiology. Indeed, T3 is required for the transcriptional activation of mitochondrial genes leading to increased retinal mitochondrial respiration and ATP production [5]. In addition, THs regulate photoreceptor function and Müller-glia-coordinated intercellular communication involved in light/dark adaptation [5].

Like in all other TH-responsive tissues, in the retina, the time and cellular-specific customization of TH signaling is determined by an exclusive endowment of different isoforms of TH transporters, TH plasmatic and nuclear receptors, and TH-processing enzymes, which shape intracellular TH availability and specific genomic and non-genomic TH activities [10].

Different grades of TH dyshomeostasis, ranging from overt or subclinical thyroid disorders to minor alterations in the euthyroid range, are frequently observed in patients with retinal sight-threatening diseases such as diabetic retinopathy (DR) and age-related macular degeneration (AMD) [11,12,13,14,15]. Conclusive demonstration of causality between TH alterations and retinal disease is still lacking, as is the knowledge of putative mechanisms whereby altered TH signaling might impact retinal disease. However, the available preclinical results suggest that a long-lasting reduction in TH signaling might favor DR progression [16], while in AMD, where a positive association with free thyroxine (FT4) circulating levels has been reported [15], increased TH signaling may be detrimental. More studies are required to reconcile these apparent counterintuitive results.

In this review, we provide novel insights on TH signaling relevant to retina development and function and highlight emerging findings linking altered TH homeostasis to DR and AMD. Remaining knowledge gaps on this connection are also discussed. An improved comprehension of the role played by TH signaling in retinal health and disease might pave the way for future studies aimed at the identification of novel strategies for DR and AMD prevention and management.

## 2. TH Signaling

The hypothalamus/pituitary/thyroid axis controls thyroidal secretion of thyroxine (T4) and T3 for constant regulation of metabolic processes [17]. Briefly, the hypothalamus secretes thyrotropin-releasing hormone (TRH), which promotes thyroid-stimulating hormone (TSH) release by the anterior pituitary gland. TSH mainly regulates the synthesis and secretion of TH by interacting with TSH receptors (TSHRs) on thyroid follicular cells [17]. High serum TH concentrations negatively feedback on TRH and TSHR expression to maintain homeostatic levels [18]. Circulating T4 and T3 are transported to peripheral tissues by thyroxine-binding globulin or transthyretin. Then, membrane transport proteins, including monocarboxylate transporters (MCT) 8 and 10 (also known as Slc16a2 and Slc16a10, respectively), bind to and transport TH into the cytoplasm. The thyroid gland produces and secretes a greater amount of the prohormone T4 (about 80%) than the biologically more active T3 (about 20%). Then, the extrathyroidal monodeiodination of T4 and T3 by deiodinase enzymes balances the levels of active and inactive THs available to different cell types and tissues, thus ensuring an optimal fine-tuning of TH action [10,19,20,21].

T4 is converted to T3 by the type 1 and 2 iodothyronine deiodinases (DIO1, DIO2). DIO1-mediated deiodination of T4, occurring mainly in the liver and skeletal muscle, is the source of most of the circulating T3, while DIO2 regulates the intracellular bioavailability of the hormone [22,23]. Intracellular T4 and T3 can be converted by type 3 iodothyronine deiodinase (DIO3) into the inactive reverse T3 (rT3) and 3,3′-diiodothyronine (T2), respectively [19,24]. When inside the cell, T3 translocates to the nucleus where it binds to TH receptors (THRs) and fosters transcriptional regulation.

THRs belong to the nuclear hormone receptor superfamily of transcription factors [25,26,27]. THRα1, THRβ1, and THRβ2, the most studied isoforms of THR, are characterized by identical DNA-binding and T3-binding domains but divergent amino termini [26,27]. THRα2, predominantly detected in the central nervous tissue and the heart, lacks a T3 binding domain and may function to inhibit THR transactivation by competing for the same DNA-binding elements [28,29]. THRs bind to DNA at sites called TH response elements (TREs). TH-dependent regulation of transcription is generally described with a model of a chromatin folding switch [30]. On T3 positively regulated target genes, unliganded THRs recruit co-repressors, thus favoring a closed chromatin conformation [31,32]. T3 binding induces a conformational change in THR, leading to detachment of corepressors, binding with coactivators, and increased chromatin accessibility [31,32]. The main relevant physiological implication of such a mechanism is that at low TH concentrations, as in the presence of hypothyroidism, or a low T3 state, the unliganded THRs might suppress transcription instead of remaining as inactive, passive receptors. In line with this interpretation, hypothyroidism models present a much more severe phenotype than THRα and THRβ double-knockout mice [33,34,35].

It has been proposed that at negatively regulated TH targets, corepressors and coactivators act in an opposite manner with respect to positively regulated genes [36]; however, the underlying mechanisms are not completely understood. In addition, recent evidence suggests that different DNA-binding motifs might be preferentially enriched at T3-activated or repressed target sites, or that T3-facilitated gene repression might be due to reduced affinity of liganded THR for chromatin [30,37].

Besides their binding to THRs, T3 and T4 can also interact with the extracellular domain of the plasma membrane protein integrin αvβ3 and induce intracellular signal transduction. Through such a mechanism, TH signaling may control cellular processes, including proliferation, apoptosis, intracellular protein trafficking, and phosphorylation/activation of THRs [38]. In vitro evidence indicates that T4 has a higher affinity for integrin and higher activity at the receptor than T3 [39,40].

## 3. Role of Thyroid Hormone Signaling in Retinal Development and Function

The mature retina comprises several neuronal cell types (rod and cone photoreceptors, bipolar, horizontal, amacrine, and ganglion cells), Müller glial cells, and pigment epithelial cells [41,42]. Rods and cones, the main types of photoreceptors, are responsible for dim light vision and daytime vision, respectively [43,44,45]. In these cells, opsin proteins initiate the phototransduction cascade converting light signals into neural signals to be passed to bipolar cells and then to ganglion cells, which, in turn, transmit visual information to the brain via the optic nerve. Different kinds of opsin proteins define three subtypes of specialized cone photoreceptors: blue/S cones express short-wavelength-sensitive S opsin, green/M cones express medium-wavelength-sensitive M opsin, and red/L cones express long-wavelength-sensitive L opsin [44,46,47]. Studies in mice have demonstrated that S opsin and M opsin form opposing, overlapping gradients along the dorsal–ventral axis, with S opsin being expressed more in the ventral region, which may provide an anatomical base for color vision [48].

**Figure 1:**
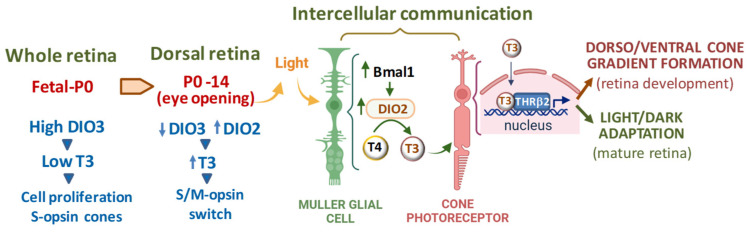
Role of TH signaling in retinal development, postnatal maturation, and light/dark adaptation. Bmal1: Basic helix-loop-helix ARNT-like protein 1; DIO2: Type 2 iodothyronine deiodinase; DIO3: Type 3 iodothyronine deiodinase; THRB2: Thyroid hormone receptor beta 2. Image produced in “Biorender.com”. Upward and downward arrows next to acronyms indicates, respectively, increased or decreased gene expression or hormone level.

Several studies have highlighted the pivotal role of TH signaling in retina maturation by promoting the prenatal cone precursor proliferation and the post-natal photoreceptor differentiation with M opsin expression, and formation of the dorsal–ventral cone gradient. During fetal life till birth (P0), THs are evenly distributed at low concentrations throughout the retina, which is associated with cell growth and default S opsin expression [49,50] (Figure 1). TH levels start increasing at P0 until, by post-natal day 10 (P10), higher levels of THs are found in the dorsal retina and lower levels are found in the ventral retina, suggesting a role of TH in promoting M opsin expression in the dorsal retina and S opsin expression in the ventral retina [49]. Consistent with these findings, an increase in S opsin and a decrease in M opsin were observed in genetically induced hypothyroid mice [51,52]. On the contrary, when mouse embryonic retinas were treated ex vivo with exogenous T3, fewer S-opsin-positive cones and more M-opsin-positive cones were observed [49].

In addition to rodents, changes in TH levels also affect retina development and cone subtype specification in fishes, chickens, and humans, suggesting the importance of such regulation as a conserved process across vertebrate species [4].

The spatio-temporal changes in retinal TH concentrations depend on the activity of DIO3 and DIO2 (Figure 1). Early in retinal development, high DIO3 expression in the whole organ determines low T3 concentrations that allow retinal growth and S cone specification. Late in retinal maturation, DIO2 expression prevails in the dorsal retina, thus increasing regional T3 levels, favoring L/M cone specification while repressing S cones [50]. As observed in the maturation/regeneration of other mammalian organs, including heart, liver, and skeletal muscle [11,53], it is conceivable that inhibiting T3 signaling via DIO3 activity drives the first phase of cone progenitor cell proliferation in embryonic life, while induction of DIO2 in the post-natal stages of organ development enhances T3 availability to prompt cell differentiation. The observation that DIO3 deletion in mice leads to cone-selective death demonstrates the detrimental effect of a premature increase in T3 levels in the developing retina [54]. Although the exact mechanisms controlling the DIO3/DIO2 switch in vertebrate retina maturation are still poorly understood [55], it has been demonstrated that the circadian clock gene Basic helix-loop-helix ARNT-like protein 1 (Bmal1) plays a central role in inducing the upregulation of DIO2 necessary for the establishment of the dorsal–ventral gradient of S and M opsins [56]. Besides being involved in retinal development, this process seems to also be important in the circadian oscillation of retinal DIO2 levels and T3 availability that influence photoreceptor function in the mature retina, which is consistent with a continual role for TH signaling in cone maintenance in adults [56].

In this regard, a recent paper unveiled a pivotal involvement of TH signaling in mediating adaptation to light conditions in the adult retina [5] (Figure 1). As revealed by single-cell RNA sequencing, other retinal cell types, in addition to photoreceptors, show TH-regulated dynamic molecular changes and enriched pathways under light/dark adaptation [5]. In particular, it has been demonstrated that light enhances the transcription of DIO2 in Müller cells, thus increasing the level of T3 available for adjacent retinal cells. The resultant modulation of mitochondrial function and gene expression is responsible for adjusting the light responsiveness of cones to light conditions. Such adaptation in cones is significantly impaired by a loss of DIO2 specifically in Müller cells, indicating these glial cells as master regulators of retinal intercellular communication [5].

TH receptors also contribute to cell-specific customization of TH action. THRβ2 is the main THR isoform expressed in cone photoreceptors and is responsible for the T3-dependent specification of cone function [57,58]. In murine models, deletions or mutations in the THRβ2 coding sequence induce a complete loss of M opsin and an increase in S opsin expression, resulting in a form of blue monochromatic color blindness [49,50,59], while targeted mutations in the noncoding enhancer sequence within the *THRβ2* gene greatly impair M opsin expression and sensitivity to long wavelengths [57]. It has been shown that a homodimer THRβ2 form is required to activate M opsin expression, while a heterodimer with retinoid X receptor-γ (RXRγ) inactivates S opsin expression [49,59]. Consistent with these observations, loss of RXRγ has been reported to induce S opsin expression in the dorsal retina without affecting M opsin transcription [49].

**Figure 2:**
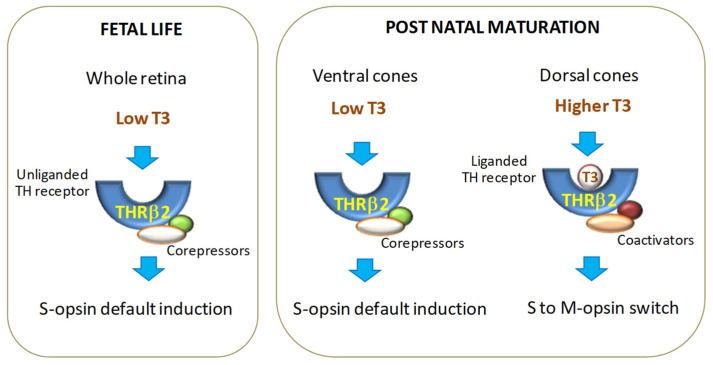
Proposed model for the spatio-temporal regulation of cone precursor proliferation and cone opsin specification by unliganded and liganded THRβ2.

In the human retina, THRβ2 has been shown to have a similar role as in rodents. Its disruption affects photoreceptor differentiation and function, as observed in patients with resistance to TH syndrome [60,61]. During retinal development, THRβ2 shows a temporal dynamic expression [59,62,63,64], with a first peak concomitant with the end of cone precursor generation and a second phase during the shift from S to M opsin activation [49,63]. Given the low level of T3 concentrations in the early phase of retinal maturation, and considering that THRβ2-dependent regulation of gene expression depends on the binding of cofactors [25], it can be speculated that, at this time point, the recruitment of corepressors by the unliganded THRβ2 prevents premature cone specification. Indeed, based on the classical THR mode of action [31], THRβ2 mutations blocking coactivator binding decrease M opsin levels, while it has no effect on S opsin expression. Instead, THRβ2 mutations causing either constitutive binding of a corepressor or disruption of T3 binding result in an increase in S opsin expression and a loss of M opsin expression in the dorsal retina [65]. Other THRβ2 mutations that prevent ligand binding, but are otherwise functional, result in the loss of all M cones and induction of S cones throughout the retina [49], suggesting that the corepressor binding activity and chromatin remodeling drives the S vs. M opsin decision [49,65] (Figure 2).

The remarkable ability of THRβ2 to prompt counter-gradients of S and M opsins in mice [59,64] led Aramaki et al. [66] to postulate a wider role of the receptor in cone diversity specification through differential activation/repression of groups of genes in a dorsal–ventral pattern. Using single-cell transcriptomic analyses of the superior (dorsal) and inferior (ventral) mouse retina at different postnatal developmental ages, from P2 to adulthood, the authors demonstrated the emergence of cone subtypes between P8 and P21, after eye-opening, in concomitance with the appearance of superior and inferior gradients of gene expression under the control of THRβ2 [66]. These data are in line with previous findings suggesting the pivotal effect of light exposure in the postnatal maturation of photoreceptors mediated by TH signaling [67].

In addition to its role in cone opsin specification, THRβ2 also regulates cone survival. For example, excess T3 signaling induces cone apoptosis through THRβ2, as observed in DIO3^-/-^ mutant mice, presenting with increased TH serum levels [54,68,69].

In contrast to THRβ2, THRβ1 peaks later during retinal maturation. Its expression is not restricted to cones, but it is also localized in amacrine cells and ganglion cells [70]. TRβ1-knockout mice displayed only minor changes in opsin expression and normal electroretinographic responses, suggesting a minor involvement of TRβ1 in the regulation of photoreceptor function. On the other hand, the identification of THRβ1 transcript in the retinal pigment epithelium, in the ciliary margin zone, ciliary body, and iris points to a major role of the receptor in non-neural eye tissues [70].

THs also have a role in the normal development of retinal cytoarchitecture [71]. For instance, hypothyroid rats showed a decrease in eyeball volume and thinning of the retinal layers, along with reduced transverse dimension and retarded myelination of the optic nerve [7]. These animals also had lower levels of sirtuin 2 (SIRT-2) protein in the ganglion cell layer, suggesting a possible action of TH mediated by the induction of SIRT-2 signaling [71].

Finally, studies have assessed the impact of TH on the development of retinal vasculature. In two population-based studies, TSH levels within the upper range or above were associated with retinal arteriolar narrowing [72,73]. Hypothyroidism induction in rat pups resulted in retarded retinal vessel development and pre-retinal neovascularization resembling retinopathy of prematurity (ROP). In this experimental setting, reduced TH signaling might have caused a state of relative retinal hypoxia, thus favoring pro-angiogenic responses and new vessel formation [74]. Decreased retinal vascular density in neonatal mice was observed after administration of high T4 doses [75], while no effects were reported with more physiological T4 concentrations either in normal rats or in an ROP model [75,76,77]. All the above findings are consistent with a recent paper based on the Rotterdam study involving 5142 middle-aged and elderly participants, in which high and low levels of FT4 were associated with lower global brain perfusion and narrower retinal vessels than middle levels of FT4 [73].

Collectively, the maintenance of TH homeostasis seems to be critical both for retinal development and mature retina physiology, which may be of relevance, especially in the context of retinal pathologies where altered TH levels have been reported.

## 4. TH Signaling in Diabetic Retinopathy

### 4.1. DR: Disease Overview

Diabetic retinopathy (DR) is a severe sight-threatening complication of diabetes mellitus of both type 1 and 2, classically attributed to the noxious effects of chronic hyperglycemia on retinal microvessels [78,79,80]. Diabetic retinal disease is classified as either non-proliferative DR (non-PDR) or proliferative DR (PDR) [80]. Early non-PDR is characterized by microvascular lesions and fluid leakage into the retina. Over time, inadequate oxygenation of the retina causes the release of growth factors such as insulin-like growth factor 1 and vascular endothelial growth factor (VEGF) [81], determining the progression from non-PDR to PDR [81] characterized by the growth of new, abnormal blood vessels. These vessels develop in different parts of the retina, resulting in blinding complications, such as neovascular glaucoma, with damage to the optic nerve. Moreover, the formation of fibrovascular scar tissue can induce tractional retinal detachment [82,83,84,85].

DR is a multifactorial disease in which different pathways ultimately cause oxidative stress and inflammation [86]. The key factor involved in vascular pathology, from microvascular damage to neoangiogenesis, is VEGF [87]. Different cells are associated with the pathogenesis of DR, including endothelial, Müller, ganglion, and pigment epithelial cells [88,89]. In particular, Müller cells express and secrete growth factors and cytokines that lead to retinal neuron and capillary cell dysfunction associated with diabetic epiretinal membrane formation [90,91,92,93]. In addition, Müller cells may exhibit characteristics of fibroblast cells that, in response to cytokines, generate traction forces [91,94]. Although DR has been considered a purely vascular pathology, it is suggested that neurons, the most fragile and demanding cells in the retina, are the first to be affected by oxidative stress and inflammation [95,96]. Consistent with this hypothesis, a state of neuroretinal degeneration, mainly characterized by retinal ganglion cell depletion and decreased thickness of the nerve fiber layer, has been reported both in patients and in animal models [97], and retinal ganglion cell functional and structural damage seem to precede the microvascular changes [95]. The discovery of neuroprotective properties of VEGF [98,99], together with evidence indicating VEGF upregulation induced by oxidative stress [99,100], supports the hypothesis that, during diabetes, VEGF is induced in an attempt to protect the retinal neurons and is then progressively upregulated, leading to early vascular damage and late vascular proliferation. Based on this hypothesis, antioxidant or neuroprotective treatments prevent VEGF upregulation and vascular damage [101]. This chain of events suggests that in the retina, as in other organs and tissues, cells exposed to diabetic stress implement a series of compensatory mechanisms attempting to face the suffering condition before displaying overt degeneration. VEGF may be one of such signals released by the retina in the early phases of DR, but other events may be activated in the context of a protective strategy. Similar to VEGF upregulation, these other events may confer protection in an early phase but they may turn detrimental in the long run. An understanding of these mechanisms may reveal unexpected avenues for the treatment of the disease.

### 4.2. Impact of TH Signaling on DR: Clinical Evidence

Several observational clinical studies have assessed the role of reduced TH signaling in type 2 diabetes mellitus (T2DM) and microvascular complications [14,102,103,104]. A correlation between low thyroid function and DR has been observed both in overt and subclinical hypothyroidism [105,106,107,108,109]. Hypothyroid T2DM patients treated with T4 replacement therapy were exposed to a significantly lower risk of developing DR compared to untreated subjects [110]. Subclinical hypothyroidism is more prevalent in T2DM than in the general population and is associated with an increased risk of developing DR [12,106]. Higher circulating TSH concentrations are indicated as an independent risk factor for DR in T2DM patients and are paralleled by retinal arteriolar narrowing, lower arteriovenous index, and altered VEFG production [12,72,109,111]. Noteworthy, in euthyroid subjects with T2DM, FT3 and FT4 levels are negatively associated with retinal microangiopathy [102]. In addition, TH serum concentrations in the lowest range and a reduction of central and peripheral TH sensitivity have been associated with retinal microvascular pathology and indicated as independent risk factors for DR [13,14,112].

The overall results suggest that even mild alterations in TH homeostasis may adversely impact DR evolution, which is in line with previous observations in other pathological conditions such as heart failure and post-ischemic heart disease [113,114,115,116].

Some studies have also assessed the occurrence of thyroid dyshomeostasis in patients with type 1 DM (T1DM) and its impact on retinal microangiopathy complications. In a study conducted in a Brazilian population, TSH values between 0.4 and 2.5 mU/L were associated with a lower risk of developing DR and renal failure compared with patients with higher TSH levels [117]. In adult euthyroid people with T1DM, higher FT3 concentration was associated with better metabolic control of the disease and lower prevalence of retinal microvascular alterations [118]. T1DM has also been associated with autoimmune thyroid disease [119,120], and the co-occurrence of T1DM and autoimmune thyroiditis in children has been shown to worsen the status of retinal parameters [121]; even though further studies are necessary to confirm the potential influence of this observation on the occurrence of DR. Contrasting results have also been reported. In a small study of adult patients with T1DM, the positivity to antibodies against thyroid peroxidase, thyroglobulin, or thyroid-stimulating hormone receptor was associated with less severe retinal microangiopathy [122]. A successive retrospective study confirmed that patients with TDM1 and autoimmune thyroiditis were metabolically better balanced and were significantly less likely to have non-PDR than the control group, suggesting that autoimmune polyglandular syndrome could have a protective effect on the development of DR [78]. The reason for the conflicting results is still unclear. Although differences in age or study design might have played a role, future investigations in a larger population are necessary to definitively state the protective or detrimental role of thyroid autoimmunity in the development of DR in T1DM subjects.

### 4.3. Impact of TH Signaling on DR: Experimental Evidence

Several in vivo and in vitro experimental observations corroborate the role of altered TH signaling in DR evolution.

Experiments using the streptozotocin (STZ) rat model of T1DM showed a correlation between the progressive decrease in plasma FT4 and FT3 concentrations and a thickening of the capillary basement membrane [78,123]. Another work using either a Zucker diabetic fatty rat model of T2DM or an STZ rat model of T1DM reported a connection between reduced FT4 levels and the development of cone vision deficiency characterized by photoreceptor outer segment degeneration and an increase in the number of retinal dual cones (that is, cones expressing S and M opsins simultaneously) [124]. These data are in line with previous findings demonstrating that inadequate TH levels may lead to the appearance of dual cones or to an increase in the number of S cones, even in adult rats [52].

In a db/db mouse model of T2DM, typical functional traits and molecular signatures of DR were paralleled by a tissue-restricted reduction in T4 and T3. This local condition of low T3 has been ascribed to DIO3 upregulation accompanied by a reduction in DIO2 and TH receptor expression. Concurrently, T3-responsive genes, including mitochondrial markers and microRNAs (*miR-133-3p*, *338-3p,* and *29c-3p*), were expressed at lower levels [16]. The underlying mechanism was investigated in Müller cell cultures, and the results indicated that oxidative stress, imposed by high glucose concentrations, leads to DIO3 upregulation through a nuclear factor erythroid 2-related factor 2/hypoxia-inducible factor-1 pathway. The consequent drop in T3 levels would drive the downregulation of the DIO3 inhibitor *miR-133*-*3p* leading to further DIO3 upregulation and exacerbating the low T3 state. These in vivo and in vitro observations permit the hypothesis that while reduced TH signaling may represent an initial adaptive response to the high glucose-induced oxidative stress, in the long run, it may increase cell vulnerability due to persistent mitochondrial dysfunction [16].

A more recent paper confirmed the presence of decreased retinal DIO2 gene and protein expression in STZ mice [125]. Furthermore, when cultured in high glucose, retinal astrocytes and microvascular endothelial cells showed a significant reduction in DIO2 protein and T3 production, in association with increased markers of inflammation and cell death. These alterations were prevented by T3 but not T4 supplementation [125], suggesting a major role of the genomic pathway in the observed protective effect of T3.

TH homeostasis also impacts pericyte (PC) survival. One of the main functions of PCs is to contribute to the integrity of the inner blood–retina barrier; therefore, PC loss may facilitate capillary instability and vascular leakage, which in turn favors microaneurysms and angiogenesis [126]. In relation to the higher prevalence and more severe form of DR in patients with subclinical hypothyroidism, interesting research demonstrated the role of TSH/TSH-receptor (TSHR) signaling activation in worsening high glucose-induced intrinsic cell death in human PCs through the elevation of ROS levels and mitochondrial dysfunction [111]. As observed in other tissues and cell types, including hair follicles, liver, fat, erythrocytes, and endothelial cells [127,128,129,130,131], a TSH/TSHR1 signaling cascade may directly impact the physiology/survival of PCs, independent of TH levels. The finding of functional TSHR on PCs is of particular clinical relevance given that loss of PCs is the predominant pathological change observed in early DR and precedes the occurrence of diabetic vascular alterations [107,132].

## 5. TH Signaling in Age-Related Macular Degeneration

### 5.1. AMD: Disease Overview

Age-related macular degeneration (AMD) is a complex eye disorder and the leading cause of incurable blindness in the elderly worldwide. It affects one in eight 60 years or older people, and, according to a recent estimate, by 2040 the global burden of AMD will rise to close to 300 million [133].

Clinically, AMD is characterized by late-onset progressive neurodegeneration of photoreceptors and RPE at the macula, the central region of the retina responsible for keen central vision [134]. AMD is classified into two different phenotypic categories: non-neovascular (dry; nonexudative), and neovascular (wet; exudative). The dry form of AMD is characterized by extracellular deposition of lipid or protein material called drusen at the level of the RPE accompanied by RPE hyperpigmentation and atrophy. In the advanced stage of dry AMD, the atrophic areas become confluent, a condition termed geographic atrophy. Wet AMD is another advanced form of AMD caused by abnormal growth of blood vessels beneath the neurosensory retina or RPE [135]. Although the neovascular form affects only 10% of AMD patients, it is responsible for about 90% of AMD-related vision loss [136]. A typical feature of AMD is excessive vascular permeability associated with immune cell infiltration [137]. Despite the major contribution of microvascular alterations to wet AMD progression, about 1/3 of patients do not benefit from intravitreal injections of VEGF inhibitors due to conversion to a fibrovascular disorder [137,138,139]. After new vessels are formed, several cell types, including choroidal fibroblasts, macrophages, and pericytes, can be activated/transdifferentiated in myofibroblasts. Thereafter, excessive production of extracellular matrix proteins such as collagen, fibronectin, and laminin triggers macular fibrosis and retinal degeneration [140]. In addition, the formation of fibrovascular membranes at the vitreoretinal interface may result in vitreous hemorrhages and/or tractional retinal detachment [138]. A better understanding of the mechanisms and molecular cues responsible for the transition from neovascular to fibrotic phenotype may help in the development of novel interventions targeting fundamental aging processes involved in the aggressive form of AMD.

### 5.2. Impact of TH Signaling in AMD: Clinical Evidence

Thyroid dyshomeostasis has been analyzed in relation to increased risk of developing AMD [141,142,143]. A study of a large population-based sample of 50-year-old adults suggested a correlation between hypothyroidism and AMD, especially in white women [141]. A successive work demonstrated that overt hyperthyroidism in older patients was also independently associated with a three-fold increase in the risk of AMD development [142]. The above findings were confirmed by more recent data showing that individuals with prior hyperthyroidism or hypothyroidism have, respectively, eight-fold and three-fold higher risks of developing AMD later in their lives [143]. Regarding the euthyroid condition, different from DR, a positive association between levels of FT4 and AMD was reported by the prospective population-based Rotterdam study performed on approximately 15,000 subjects recruited over more than two decades [15]. Increased FT4 serum levels in the presence of unaltered T3 and TSH levels were confirmed in euthyroid patients with wet-type AMD [144]. Furthermore, a genome-wide association study of 72,167 individuals showed that genetic variants predisposing to higher FT4 levels within the normal range were at higher risk of AMD [145]. An association between the development of geographic atrophy and therapeutic supplementation of TH was detected by the AREDS study [146]. However, any link between the use of thyroid medications and AMD development was not confirmed by a recent meta-analysis [147]. Further studies are required to understand the putative causal relationship and the underlying mechanisms linking increased FT4 availability and AMD development. T4 may promote angiogenesis through the so-called non-genomic effect exerted via binding to integrins on the membrane of endothelial cells [148,149]. Integrin receptors αVβ3, αVβ5, and αV5β1 are expressed in the retina and are associated with choroidal angiogenesis observed in dry and wet AMD [150]. It is conceivable that higher FT4 levels might favor neoangiogenesis, as observed in AMD, through the integrin cascade.

### 5.3. Impact of TH Signaling in AMD: Experimental Evidence

In light of the clinical data linking higher free serum FT4 levels to increased risk of AMD, recent studies have investigated the effects of TH signaling inhibition on RPE and photoreceptor survival in a chemically induced mouse model of AMD [69,151].

In AMD models, treatment with the anti-thyroid agent methymazole or THR deficiency almost completely preserved RPE and photoreceptors from damage or cell death, reversed gene expression alterations, and partially preserved retinal function [69,151]. The involvement of THR subtypes varied in the RPE and retina. Deletion of THRα1 or THRβ protected RPE, rods, and cones, whereas deletion of THRβ2 protected RPE and cones but not rods [152], confirming the non-redundant role of the various receptor protein isoforms. Biochemical/gene expression analyses showed that photoreceptor degeneration induced by excess TH likely involves multiple cellular mechanisms, including oxidative stress, necroptosis, and inflammation [153]. Oxidative damage, inflammatory lesions, and cell death are typical features of cone and rod degeneration linking AMD to inherited forms of retinal dystrophies, such as retinitis pigmentosa, Leber congenital amaurosis (LCA), and cone–rod dystrophies [153]. These characteristics are suggestive of common underlying TH-related mechanisms controlling cellular degeneration/death processes that may be targeted to reduce photoreceptor loss, regardless of the origins of the disease. In this regard, several studies have shown a link between excess TH signaling and photoreceptor degeneration in mice models of hereditary retina dystrophies such as LCA and cone dystrophy/achromatopsia. In these models, similar to what is observed in AMD, suppression of TH activity via inhibition of DIO2, overexpression of DIO3, or deletion of TH receptors reduces photoreceptor degeneration [153,154], suggesting that intraretinal repression of TH signaling could be a protective strategy against both AMD and inherited retinal dystrophy.

## 6. Conclusions and Future Perspectives

**Figure 3:**
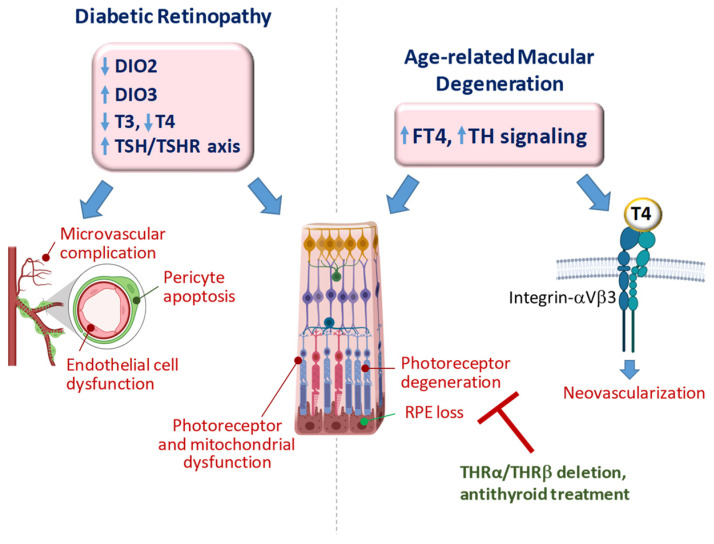
Proposed implications of altered TH signaling in DR and AMD based on clinical and experimental evidence. Upward and downward arrows next to acronyms indicate, respectively, increased or decreased gene expression, hormone levels or signaling activation.

TH signaling orchestrates retinal development and function. According to the state-of-the-art, unbalanced TH homeostasis may play a role in the etiology/pathogenesis of DR and AMD (Figure 3).

While the long-lasting reduction in T3 signaling has been associated with DR progression, even mild rises in FT4 are linked to an increased risk of developing AMD. Although the mechanisms underlying such apparently opposite behavior of THs in different pathological settings still need to be unveiled, it has been speculated that a protracted low T3 state in the diabetic retina might favor the mitochondrial-dependent death of neurons [16]. On the other hand, increased T4 signaling in AMD might prompt new vessel formation or interfere with cellular functions of RPE, such as renewal of photoreceptor cell outer segments or maintenance of retinal adhesion. It is conceivable that in wet AMD, noxious signaling initiated by FT4 at integrin αvβ3 might play a prominent role, especially considering the high affinity of this hormone for the plasma membrane receptor. It remains to be determined if this regulatory circuit is also implicated in dry AMD.

Whatever the pathways, preclinical data provide a glimpse into possible therapeutic strategies exploiting TH signaling. If a DIO3-driven retinal low T3 state is confirmed in patients with DR, local T3 replacement or DIO3 targeting could represent an option to be tested in future preclinical and clinical studies. On the contrary, AMD conditions could benefit from T4 antagonism. For example, tetraiodothyroacetic acid, a deaminated analog of T4 that can block the activity of TH on integrin αVβ3, has been shown to inhibit the pro-angiogenic effect of both VEGF and erythropoietin in retinal endothelial cells [155] and might be tested as a viable therapeutic strategy against neoangiogenesis-dependent retinal diseases. However, it should be emphasized that the pathogenic mechanisms underlying DR and AMD are different, and therefore may interact differentially with the local levels of THs and TH signaling in determining conditions of protection and/or induction–aggravation of the pathology. Studying these crosstalking mechanisms could indicate more clearly which therapeutic approach will be preferable, in which phase of the disease, and for which specific clinical condition.

In conclusion, the involvement of TH signaling in complex diseases such as DR or AMD is a promising field that is still largely unexplored. Hopefully, studies in the near future will lead to the identification of novel TH-based strategies for the prevention and management of retinal disorders.

## Data Availability

Not applicable.
